# Outcomes of allergy testing after an emergency department visit for food anaphylaxis

**DOI:** 10.1186/2045-7022-1-S1-P46

**Published:** 2011-08-12

**Authors:** Ronna Campbell, Wyatt Decker

**Affiliations:** 1 Mayo Clinic, Emergency Medicine, Rochester, USA

## Background

Anaphylaxis is a potentially life threatening allergic reaction. Failure to identify the inciting allergen places patients at risk of future life-threatening allergen exposure. Anaphylaxis guidelines recommend that all patients who experience anaphylaxis from an allergen encountered in a non-medical setting carry self injectable epinephrine and follow up with an allergist. Very little data is available on outcomes of allergy follow up after an emergency department (ED) visit for anaphylaxis. The aim of our study was to determine the outcomes of allergy follow up after an ED visit for food anaphylaxis.

## Methods

A retrospective cohort study was conducted in an ED setting with approximately 80,000 visits per year. Patients presenting to the ED for anaphylaxis and allergic reactions were screened from April 2008 to June 2010 and all patients who fulfilled NIAID/FAAN criteria for anaphylaxis and provided consent were included. A standardized data abstraction form was used to collect data.

## Results

Two hundred and twenty patients constituted the study sample and were included in the study. The median age was 33.5 years (IQR 18.8-49.6) years. One hundred and twenty-eight (58.2%) were females. Suspected allergens in the ED were food in 79 (35.9%), medication in 47 (21.4%), insect venom in 27 (12.3%), others in 24 (10.9%) and unknown in 43 (19.5%) patients. A total of 81 patients (36.8 %) followed up with an allergist. Among patients with food anaphylaxis, 40 (50.6%) followed up with an allergist of which 34 (85%) had allergy testing. Among the 34 patients, skin testing was performed on 16 (47.1%), specific IgE antibody tests in 22 (64.7%) and both tests in 5 (14.7%) patients. After testing, 25 (73.5%) had an allergen identified.

## Conclusions

Allergen identification and avoidance after food anaphylaxis is important in order to avoid future reactions. Our results show that most patients who followed up with an allergist underwent allergy testing and had an allergen identified. These results support current anaphylaxis guidelines.

